# Axillary Management in Clinically Node-Positive (cN1) Estrogen Receptor-Positive Breast Cancer

**DOI:** 10.3390/cancers18162574

**Published:** 2026-08-11

**Authors:** Jennifer Den, Kamil Khanipov, Vicki Suzanne Klimberg, Raj Vaghjiani

**Affiliations:** 1 Department of Surgery, The University of Texas Medical Branch, Galveston, TX 77555, USA; 2 Department of Pharmacology and Toxicology, The University of Texas Medical Branch, Galveston, TX 77555, USA

**Keywords:** breast cancer, axillary management, clinically node-positive breast cancer, axillary de-escalation, oncology

## Abstract

Background/Objectives: Axillary management in breast cancer continues to de-escalate, with trials showing similar outcomes between sentinel lymph node biopsy (SLNB) and axillary lymph node dissection (ALND) in clinically node-negative patients. However, equivalence has not been demonstrated in estrogen receptor-positive (ER+), clinically node-positive (cN1) patients. We hypothesize that SLNB would not be associated with inferior outcomes compared to ALND among this population. Methods: Using the TriNetX Network, we identified women aged ≥18 years with cN1, ER+ breast cancer who underwent axillary surgery, inclusive of both upfront surgery and neoadjuvant-treated patients. Stage IV disease was excluded. The index event was the first axillary surgery (SLNB or ALND). Propensity score matching (1:1) was performed, balancing for age, receptor status, tumor characteristics, treatment, surgical procedure, and comorbidities. Outcomes included overall survival (OS) and local recurrence (LR). Hazard ratios (HRs) with 95% confidence intervals (CIs) were calculated using Cox models. Results: A total of 1162 women were identified (SLNB 641, ALND 521). After matching, 435 patients remained in each cohort. At up to 10 years, OS was similar between groups (77% vs. 76%, HR 0.9, 95% CI 0.66–1.27). Breast recurrence occurred in 5% versus 7% (HR 0.82, 95% CI 0.47–1.42). Axillary recurrence occurred in 21% versus 24% (HR 0.9, 95% CI 0.69–1.21). None of these differences reached statistical significance. Conclusions: In ER+, cN1 node-positive breast cancer, SLNB was not associated with worse survival or recurrence compared with ALND. These real-world findings are hypothesis-generating but support the continued investigation and refinement of axillary de-escalation guidelines for clinically node-positive disease.

## Simple Summary

Surgery to remove axillary lymph nodes has long been a cornerstone of treatment for breast cancer that has spread to nearby lymph nodes. However, more extensive lymph node surgery is associated with complications such as lymphedema, pain, and reduced arm function. While recent clinical trials have supported less invasive approaches for certain patients, evidence remains limited for patients with clinically node-positive disease. In this study, we used a large multi-institutional database to compare outcomes among women with hormone receptor-positive breast cancer who underwent either sentinel lymph node biopsy or complete axillary lymph node dissection. We found no differences in long-term survival, breast cancer recurrence, or axillary recurrence between the two groups. These findings suggest that select patients with node-positive breast cancer may consider less extensive axillary surgery, supporting ongoing efforts to reduce treatment-related morbidity while maintaining effective cancer control.

## 1. Introduction

Axillary management in breast cancer has undergone dramatic de-escalation over the past two decades. Historically, axillary lymph node dissection (ALND) served as the primary method for regional staging and local control but with the introduction of sentinel lymph node biopsy (SLNB) and the maturation of multidisciplinary therapy, the need for routine ALND has been increasingly challenged.

Landmark trials such as ACOSOG Z0011, AMAROS, and others established the safety of omitting ALND in clinically node-negative (cN0) patients with limited positive nodes, achieving equivalent oncologic outcomes with reduced morbidity [1,2]. Despite this progress, these studies excluded women with clinically node-positive (cN1) disease. As a result, ALND remains common for these patients, based on the assumption that clinically positive nodes reflect higher disease burden and risk of residual axillary disease [3]. However, this standard is increasingly challenged in the era of effective systemic therapy and targeted radiation therapy.

Trials such as ACOSOG Z1071, SENTINA, and SN FNAC, have demonstrated the surgical feasibility of SLNB following nodal downstaging after neoadjuvant chemotherapy (NAC) [4,5,6]. However, these trials focus primarily on surgical accuracy and false-negative rates rather than long-term oncologic outcomes between SLNB and ALND. The ongoing TAXIS trial is the first randomized controlled trial designed specifically for cN1 ER-positive patients, defined either as detectable by palpation or by imaging, and compares tailored axillary dissection (TAD) with or without completion ALND [7]. Until the completion of TAXIS, real-world evidence may help guide clinical decisions for ER-positive (ER+), clinically node-positive patients, a group with favorable tumor biology and high responsiveness to systemic endocrine therapy [8,9,10].

The objective of our study was to use a large, multi-institutional real-world database to conduct a comparative analysis of SLNB versus ALND among women with clinically node-positive ER+ breast cancer. We evaluated a real-world propensity-score matched population of cN1 patients and hypothesized that there would be no significant differences in overall survival (OS) or local and axillary recurrence between SLNB and ALND in this population.

## 2. Materials and Methods

### 2.1. Data Source

Data used in this study were collected on 13 October 2025 from the TriNetX U.S. Collaborative Network, a global federated health research database that provides access to de-identified electronic medical records from over 140 million patients across 106 health care organizations (HCOs). Information available covers demographics, diagnoses (based on ICD-10-CM codes), procedures (coded using ICD-10-PCS or CPT), medications (coded using the RxNorm naming system, the Veterans Affairs National Formulary, or the Anatomical Therapeutic Chemical classification system), and laboratory tests. HCOs include patients with or without insurance from academic medical centers, specialty clinics, and community hospitals, representing a diverse patient population across the United States.

### 2.2. Patient Consent Statement

This retrospective study is exempt from informed consent. The data reviewed is a secondary analysis of existing data, does not involve intervention or interaction with human subjects, and is de-identified per the de-identification standard defined in Section §164.514(a) of the Health Insurance Portability and Accountability Act Privacy Rule. Data is directly retrieved from electronic health record systems of participating organizations in a systematic and standardized format.

### 2.3. Design of Cohort

We identified women 18 years or older with cN1, ER+ Stage I-III breast cancer (ICD-10-CM C50). Both HER2-positive and HER2-negative tumors were included; however, HER2 status was used for propensity score matching (PSM). Patients were stratified into two cohorts based on axillary surgery management: SLNB and ALND. The codes used for SLNB were CPT 38500, 38525, 38792, and 38900, and are the same codes used for TAD procedures. The codes for ALND were CPT 38740, 38745, 19302, 19305, 19306, 19307, and 1006917.

Importantly, before implementation of the American Joint Committee of Cancer (AJCC) 8th Edition staging system in 2018, clinical nodal staging was primarily based on physical examination, with cN1 designation reflecting palpable axillary disease. The current 8th Edition (2018) of AJCC staging system has expanded the criteria for cN1 to include either palpation or imaging-detected nodes [11]. Because the TriNetX database contains longitudinal records spanning both the pre- and post-2018 eras, our cohort likely reflects a mixture of these staging paradigms. However, the exact year of diagnosis and treatment timing are unavailable due to de-identification constraints within the TriNetX platform. Given that the dataset includes patients diagnosed before 2018 [12], a substantial proportion of the cohort was likely staged according to historical criteria, in which cN1 more commonly represented palpable axillary disease. Therefore, while our study captures a broad spectrum of cN1 disease encountered in routine practice, the overall cohort likely includes patients with a greater axillary disease burden than contemporary studies that define cN1 using imaging-based criteria.

We did not restrict cohorts to a population that received only upfront surgery or neoadjuvant systemic therapy. As an administrative database, TriNetX does not reliably distinguish the timing of systemic therapy relative to surgery; therefore, this study reflects a real-world population of cN1 patients undergoing axillary surgery, inclusive of both treatment paradigms. To address this limitation and to reflect heterogeneous practice patterns, PSM was performed on receipt of chemotherapy, radiation therapy, and endocrine therapy, ensuring comparable treatment exposure between SLNB and ALND cohorts. While treatment sequencing cannot be determined, this approach allows for balanced comparison of patients receiving similar multimodal care. It is important to note that the absence of a documented treatment code within this database should not be interpreted as the absence of treatment; rather, TriNetX reflects real-world documentation patterns and coding practices, which may differ from the completeness of registry-level treatment abstraction. A comprehensive summary of the availability and capture of relevant clinical variables within the TriNetX platform is provided in Supplementary Table S1.

Similarly, TriNetX does not reliably allow determination of whether patients undergoing initial sentinel lymph node biopsy subsequently underwent completion axillary lymph node dissection; our exposure definition represents the initial documented axillary procedure rather than a complete axillary treatment pathway. Therefore, our findings should be interpreted as an association between the initial axillary procedure documented in the medical record and subsequent outcomes.

### 2.4. Outcomes

Outcomes for our cohorts were assessed during the follow-up period 1 year after the index event (SLNB or ALND) to 10 years. The outcomes included:

- Overall survival (OS): Coded as “Deceased” under Demographics.
- Local breast recurrence: ICD-10-CM C79.81.
- Local axillary recurrence: ICD-10-CM C77.3.

In the absence of a structured recurrence variable, we selected ICD-10 codes C77.3 and C79.81 to approximate locoregional recurrence, based on their application in prior claims-based and EHR studies using administrative and registry data [13,14,15]. While these codes may capture nodal recurrence or second ipsilateral primaries, differentiating true local recurrence from a new ipsilateral primary is often not possible in retrospective datasets lacking detailed pathologic or molecular data. This limitation is further compounded by the constraints of the TriNetX platform, which does not provide granular information on surgical, pathologic, or radiation therapy. To mitigate potential misclassification from these codes, particularly the possibility that they may capture nodal disease from non-breast cancers, we additionally matched for other malignancies, including melanoma and lymphoid/lymphoma cancers, which can metastasize to the axillary nodes. We also set a minimum 12-month lag period following index surgery before recurrence codes were considered outcome events. We emphasize that these outcomes represent axillary disease-related events identified through administrative coding rather than confirmed isolated axillary recurrence.

### 2.5. Statistical Analyses

All statistical analyses were conducted using the TriNetX platform. To minimize the effect of confounding factors, we performed 1:1 PSM using a greedy nearest-neighbor algorithm with a caliper of 0.1 pooled standard deviations and without replacement. Cohorts were balanced by age, tumor stage, tumor size, HER2 and PR status, receipt of adjuvant and radiation therapy, type of breast surgery, genetic carrier status (Z14–Z15), other malignancies such as melanoma and lymphoma, and comorbidities including diabetes (E08–E13), cardiovascular disease (I20–I25), obesity (E66), and substance use (Z72.0, F10–F19). Balance was assessed by standardized difference, with <0.1 indicating an acceptable match [16].

These covariates were used for matching and adjustment purposes and are not intended to characterize the overall patient population. Procedure-related variables, including type of breast surgery, were defined using CPT codes and used for cohort assignment and covariate balancing. However, these codes may not fully capture the extent of axillary surgery and may be applied inconsistently across institutions. As such, they were not interpreted as precise indicators of operative technique.

Kaplan–Meier (KM) curves and log-rank tests were used for survival analyses. Hazard ratios (HRs) and 95% confidence intervals (CIs) were calculated using Cox proportional hazards models. Statistical significance was set at *p* < 0.05.

## 3. Results

**Figure 1:**
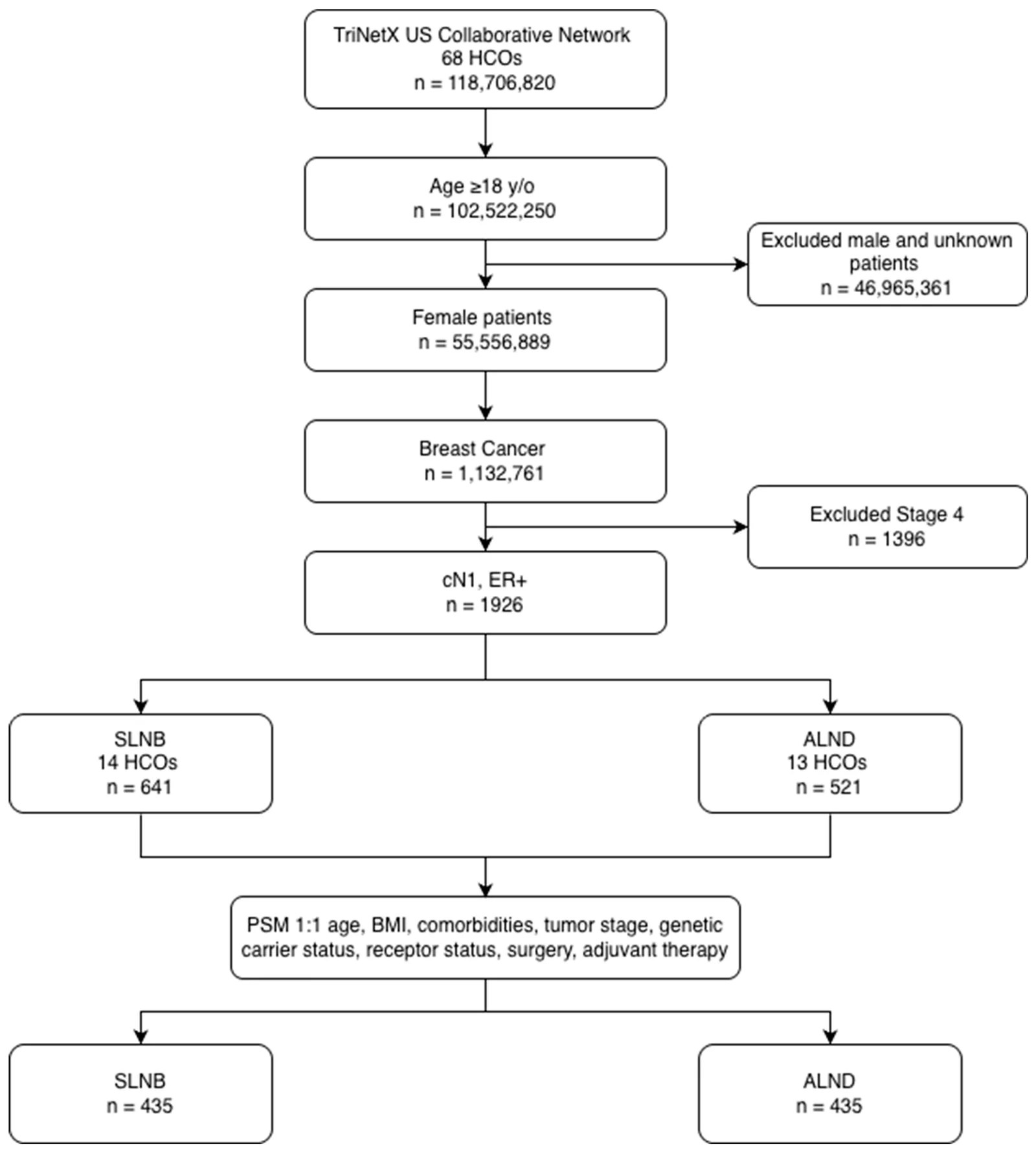
Flow diagram of cohort construction. Stage I–III female patients aged 18 years or older with cN1, ER+ breast cancer were identified, separated by SLNB vs. ALND, and propensity-matched. Because TriNetX is a dynamic, real-world database that is continuously updated as participating institutions contribute new data, patient counts may fluctuate between sequential queries and may not sum exactly across cohort selection stages.

In total, 1162 women with cN1, ER+ Stage I-III breast cancer were identified; 641 underwent SLNB and 521 underwent ALND (Figure 1). Given the dynamic structure of the TriNetX platform, cohort counts presented in Figure 1 reflect independent real-time queries rather than a single static dataset. Therefore, these values are not expected to be cumulative and may not align precisely when summed.

Prior to PSM, the SLNB group was slightly younger (mean age 54.8 vs. 56.2 years) (Table 1). Differences were observed in treatment patterns, with a higher proportion of patients in the ALND group receiving radiation therapy, while chemotherapy use was similar between groups. In addition, comorbidities were more prevalent in the ALND group (Table 1). These imbalances were largely eliminated following matching, with standardized differences reduced to <0.1 across most variables (Table 2). As described in the Methods Section, these variables were chosen specifically for matching and adjustment and are not intended to represent a complete characterization of the study population.

**Figure 2:**
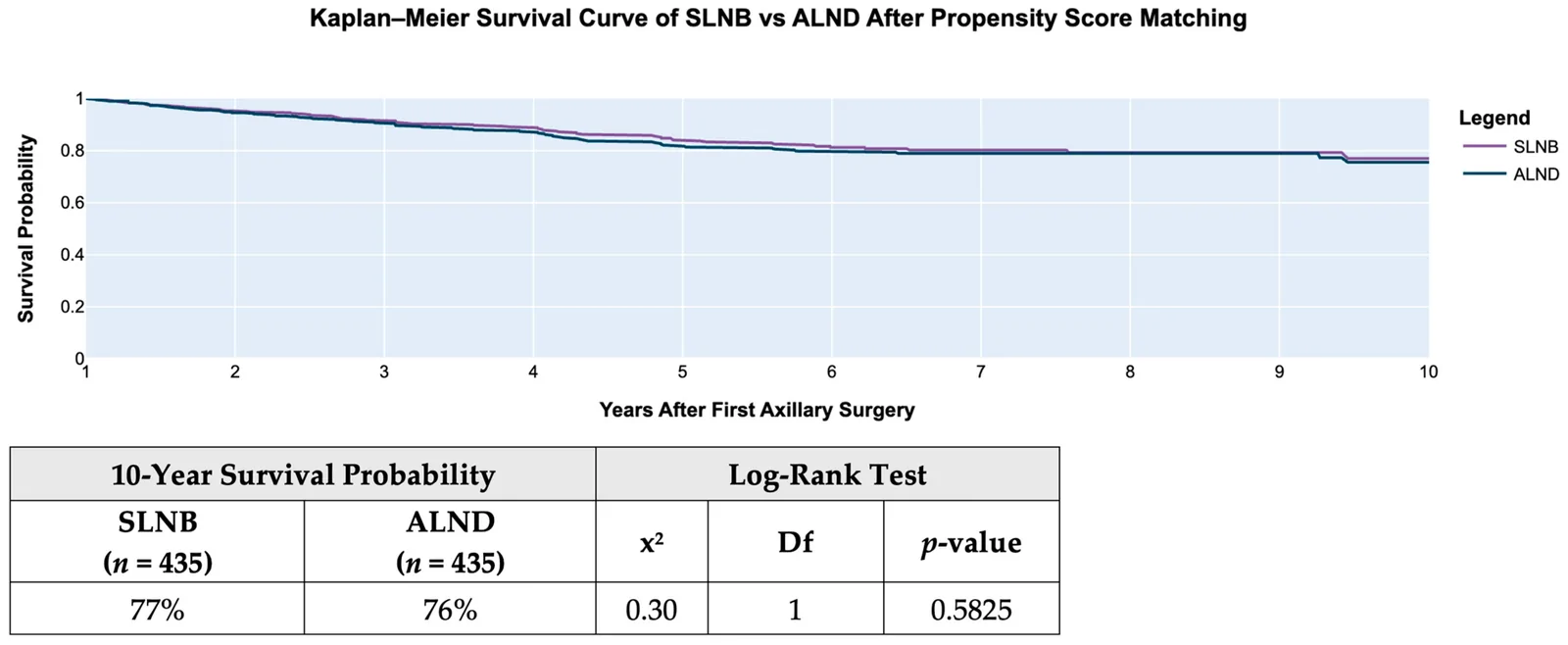
Kaplan–Meier survival curve of SLNB and ALND after PSM. Abbreviations: x^2^, chi-squared test; df, degrees of freedom.

After PSM, 435 patients remained in each cohort (Figure 1). With follow-up beginning at 1 year and extending to 10 years, OS was 77% in the SLNB group and 76% in the ALND group (HR 0.90; 95% CI 0.66–1.27) (Figure 2). Local breast recurrence occurred in 5% of patients treated with SLNB compared with 7% in those who underwent ALND (HR 0.82; 95% CI 0.47–1.42) (Table 3). Axillary recurrence occurred in 21% of the SLNB group and 24% of the ALND group (HR 0.90; 95% CI 0.69–1.21) (Table 3). There were no statistically significant differences in OS, local breast recurrence, or axillary recurrence between the two treatment groups.

## 4. Discussion

In this large, multi-institutional real-world study of women with clinically node-positive, ER+ breast cancer, omission of ALND was not associated with inferior OS or increased breast or axillary recurrence compared with standard ALND. Although observational in nature, these findings, derived from rigorously matched cohorts, add to the growing body of evidence suggesting that with modern multidisciplinary therapy, the extent of axillary surgery may be safely reduced in select node-positive patients.

As mentioned in the Methods Section, a key distinction of our study is the likely palpable nature of nodal positivity within our 10-year cohort. Prior to the AJCC 8th Edition in 2018, clinical nodal staging was largely determined by physical examination, with cN1 commonly reflecting palpable axillary nodes. Although AJCC 8 expanded this definition to include imaging-detected disease [11], real-world practice patterns and coding continue to rely heavily on clinical assessment. Given the broad time span and heterogeneous practice environments represented in TriNetX, a substantial proportion of our ER-positive cohorts likely had palpable nodal disease at presentation, and ten-year data in this study should include patients collected prior to 2018. In contrast to recent studies that include imaging-detected nodal metastases, our findings support the safety of axillary de-escalation even in patients with more overt, and potentially more advanced, axillary involvement.

The favorable outcomes observed with limited axillary surgery in our cN1, ER+ cohort are likely multifactorial. First, ER+ breast cancer is characterized by a favorable natural history and durable response to endocrine therapy [8,9,10], which may reduce the risk of locoregional recurrence and support the safety of axillary de-escalation [17]. While ER+ tumors typically show lower breast pathological complete response (pCR) after NAC [18,19], the nodal pCR rate in ER+ tumors is often higher than those observed in the breast [20,21]. Moreover, adjuvant endocrine therapy is highly effective at managing the risk of recurrence from residual disease in the breast [22,23]. Although our database cannot determine nodal response status, cohorts were well balanced for systemic therapy exposure, minimizing treatment-related confounding. This may reflect the importance of adjuvant treatment in controlling residual disease. Interestingly, despite their favorable overall prognosis, hormone receptor positive/HER2– tumors have also been identified as an independent predictor of residual axillary disease following neoadjuvant therapy [24]. This apparent paradox highlights the unique challenges of axillary management in ER+ disease and suggests that tumor biology, rather than response rates alone, should be considered when individualizing surgical management.

Second, the consistent use of radiation therapy in contemporary practice, which was also balanced between our cohorts, plays a crucial role. The AMAROS trial demonstrated that axillary radiotherapy is non-inferior to ALND, highlighting the effectiveness of radiation in managing microscopic residual disease [2]. This is further supported by evidence from a 2022 National Cancer Database (NCDB) study demonstrating that cN+, T1-T2 patients treated with SLNB and regional nodal irradiation (RNI) achieved 5-year OS rates comparable to those treated with ALND with or without RNI (88% vs. 86% vs. 78%, respectively) [25]. Our data reinforce the concept that the combination of effective systemic therapy and comprehensive axillary irradiation may reduce the incremental benefit of ALND in achieving regional control.

Finally, a recent NCDB study demonstrated that clinically positive nodal disease may overestimate true pathologic burden [26]. In this study evaluating 57,823 patients, nearly 10% of patients classified as cN1 were pathologically node-negative at surgery, and among those with confirmed nodal disease, OS did not differ significantly between patients staged as cN0 and cN1 [26]. These observations indicate that clinically and radiographically suspicious nodal disease alone may not necessarily justify routine ALND in the modern treatment era, particularly in patients with favorable tumor biology and access to systemic and radiation therapies.

Therefore, in select clinically node-positive patients, less extensive axillary surgery may be an acceptable approach to guide subsequent treatment without the morbidity associated with a complete axillary dissection. While current NCCN and ESMO guidelines continue to recommend ALND for many patients with clinically node-positive disease, our real-world analysis complements the underlying hypothesis of the TAXIS trial, suggesting that comparable long-term outcomes may be achievable with less extensive axillary surgery in carefully selected patients. Future research should focus on improving the accurate intraoperative identification of limited nodal burden and on the long-term outcomes of prospective trials to better define which patients with cN1 disease can safely avoid ALND.

We acknowledge that our observed axillary recurrence rates were substantially higher than those reported in randomized trials. Several factors likely contribute to this discrepancy. First, this disparity is a feature of using large, administrative datasets, where recurrence is captured through broad secondary disease coding. Administrative datasets rely on longitudinal ICD coding, which may capture disease progression, restaging, or delayed documentation rather than strictly defined recurrence events. Although we applied a 12-month lag period to mitigate misclassification of index disease, residual misclassification remains possible. Second, unlike a clinical trial with rigorous surveillance, protocolized treatment pathways, and carefully selected patients, TriNetX captures heterogeneous, real-world clinical practice across diverse healthcare organizations, including academic and community settings, without standardized follow-up or uniform documentation practices. As a result, outcomes may reflect variation in care delivery, surveillance intensity, and documentation timing.

Additionally, our patients likely include a population with higher baseline disease burden, as cN1 designation before 2018 was based on palpable node-positive disease, rather than imaging-detected low-volume disease. This is a critical distinction and may therefore contribute to the higher observed axillary recurrence rates. Finally, TriNetX does not distinguish isolated regional recurrence from metastatic disease involving axillary lymph nodes. These events represent coding-based surrogate outcomes and may include a broader spectrum of regional or systemic disease progression. Nonetheless, we emphasize that while absolute rates are high, the lack of a significant difference between the two surgical groups (HR 0.9, 95% CI 0.69–1.2) remains the critical finding, reinforcing that no statistically significant difference in survival or recurrence was identified between the two cohorts. Overall survival thus represents the most robust endpoint of this study, whereas recurrence outcomes should be interpreted as administrative surrogate measures rather than direct measurements of locoregional control.

Taken together, these results indicate that a less extensive coding-defined axillary surgery was not associated with a significant survival disadvantage within a matched real-world cohort of clinically node-positive ER-positive breast cancer patients. While these findings justify further prospective research into axillary de-escalation, they also underscore that modifications to standard clinical practice should be deferred until data from randomized clinical trials become available.

### Limitations

This study has several limitations inherent to retrospective analyses of large administrative datasets. While all patients were classified as cN1 at presentation, TriNetX does not provide granular data on whether nodal positivity was biopsy-confirmed or if patients were ultimately pN0 on final pathology. This reflects the “real-world” challenge where surgical planning is initiated based on clinical and radiographic presentation; however, we utilized PSM for tumor stage and size to mitigate this potential bias and ensure balanced cohorts. Furthermore, the database does not explicitly differentiate between upfront surgery and NAC, nor can it identify which patients downstaged from cN1 to ycN0 prior to surgery, both of which may influence surgical selection and recurrence risk. While we matched cohorts based on receipt of adjuvant therapy, exact sequencing and treatment details such as systemic regimens, duration of therapy, radiation fields and dosing, surgical margins, pathological nodal burden, extranodal extension, and number of lymph nodes removed with biopsy-confirmation details are not available. This lack of granular pathologic data is a recognized limitation, but it is an inherent feature of large-scale administrative datasets that capture heterogeneous, real-world clinical practice. By necessity, this study prioritizes broad generalizability across diverse clinical settings over the narrow, protocol-driven precision of randomized trials. Thus, our findings should be interpreted as reflecting heterogeneous real-world treatment pathways rather than a single standardized treatment paradigm.

Additionally, targeted axillary approaches such as TAD lack specific procedural codes, and their use is unlikely to be widely available or standardized across the community centers represented in this real-world database. Restricting the analysis only to patients treated with such specialized techniques would limit generalizability and undermine the value of a real-world dataset. For this reason, we used CPT codes for SLNB as a reproducible surrogate for de-escalated surgery. Accordingly, our analysis compares coding-defined surgical approaches rather than precisely characterized operative techniques. Finally, recurrence capture relies on secondary disease coding, which is not specific enough to reliably distinguish ipsilateral locoregional recurrence from distant spread, thus we cannot fully capture the distinction between true ipsilateral local relapse and regional or distant metastasis. Despite these limitations, our study strengths include a large, diverse, real-world patient population, rigorous PSM to balance key covariates and address potential confounders, and long follow-up of ten years. In addition, the stability of our results following PSM suggests that the lack of additional granular variables is unlikely to be the primary driver of our findings. The consistent lack of difference in both survival and recurrence across these balanced cohorts provides a robust clinical signal that warrants investigation in controlled prospective environments.

## 5. Conclusions

Our findings support the growing momentum toward individualized axillary management. In this matched real-world cohort of ER-positive clinically node-positive patients, no significant survival disadvantage was identified with less extensive axillary surgery compared with ALND. These findings are hypothesis-generating and require validation in prospective settings, but they fill a critical evidence gap regarding current axillary de-escalation patterns. As definitive data from TAXIS and other ongoing trials become available, we believe that the role of ALND in cN1 disease will likely continue to evolve toward a more personalized and less invasive approach.

## Figures and Tables

**Table 1:** Baseline PSM covariate characteristics of the study population.

	Before Matching		
Variables	SLNB (*n* = 641)	ALND (*n* = 521)	Std. Diff
**Age at index, years**			
**Mean ± SD**	54.8 ± 12.9	56.2 ± 12.4	0.1103
**Tumor size, *n* (%)**			
**T1**	613 (95.6%)	509 (7.7%)	0.1152
**T2**	261 (40.7%)	202 (38.8%)	**0.0398**
**T3**	298 (46.5%)	223 (42.8%)	**0.0742**
**T4**	83 (12.9%)	78 (15%)	**0.0584**
**Stage, *n* (%)**			
**1**	24 (3.7%)	17 (3.3%)	**0.0262**
**2**	141 (22%)	81 (15.5%)	**0.1657**
**3**	327 (51%)	246 (47.2%)	**0.0760**
**Genetic carrier**	89 (13.9%)	87 (16.7%)	**0.0782**
**Comorbidities**			
**Overweight and obesity**	95 (14.8%)	97 (18.6%)	0.1019
**Diabetes mellitus**	45 (7%)	39 (7.5%)	**0.0179**
**Cardiovascular disease**	25 (3.9%)	25 (4.8%)	**0.0441**
**Tobacco use**	153 (23.9%)	196 (37.6%)	0.3014
**Psychoactive substance use**	42 (6.6%)	23 (4.4%)	**0.0940**
**Receptor status**			
**PR positive**	168 (28.8%)	137 (29.3%)	**0.0101**
**PR negative**	337 (57.8%)	291 (62.2%)	**0.0894**
**HER2 positive**	149 (25.6%)	107 (22.9%)	**0.0629**
**HER2 negative**	352 (60.4%)	323 (69%)	0.1815
**Adjuvant therapy**			
**Anastrozole**	40 (6.2%)	44 (8.4%)	**0.0846**
**Tamoxifen**	17 (2.7%)	19 (3.6%)	**0.0570**
**Chemotherapy**	497 (77.5%)	397 (76.2%)	**0.0317**
**Radiation**	63 (9.8%)	83 (16%)	0.1829
**Surgical procedure** (CPT)			
**Partial mastectomy** (19301)	127 (19.8%)	113 (21.7%)	**0.0463**
**Partial mastectomy with lymphadenectomy** (19302)	≤10 (1.6%)	0 (0)	0.1780
**Simple mastectomy** (19303)	≤10 (1.6%)	24 (4.6%)	0.1769
**Mastectomy, subcutaneous** (19304)	≤10 (1.6%)	0 (0%)	0.1780
**Radical mastectomy** (19305)	≤10 (1.6%)	≤10 (2%)	**0.0275**
**Radical mastectomy, including internal mammary lymph nodes** (19306)	0 (0%)	0 (0%)	–
**Modified radical mastectomy** (19307)	≤10 (1.6%)	15 (2.9%)	**0.0896**

Bold font represents a standardized difference < 0.1. Abbreviations: Std. diff, standardized difference. *These variables were selected for matching and adjustment and should not be interpreted as a comprehensive description of the overall study population. Due to the longitudinal nature of the TriNetX database, variables are queried independently and are not necessarily mutually exclusive. Percentages reflect the proportion of patients with documented values for each characteristic; therefore, percentages may not sum to 100% due to missing data and incomplete capture within the electronic health record.*

**Table 2:** Baseline PSM covariate characteristics of the matched population.

	After Matching		
Variables	SLNB (*n* = 435)	ALND (*n* = 435)	Std. Diff
**Age at index, years**			
**Mean ± SD**	55.4 ± 12.4	55.7 ± 12.5	**0.0234**
**Tumor size, *n* (%)**			
**T1**	426 (97.9%)	424 (97.5%)	**0.0307**
**T2**	174 (40%)	174 (40%)	**<0.0001**
**T3**	203 (46.7%)	196 (45.1%)	**0.0323**
**T4**	69 (15.9%)	58 (13.3%)	**0.0717**
**Stage, *n* (%)**			
**1**	18 (4.1%)	15 (3.4%)	**0.0361**
**2**	77 (17.7%)	74 (17%)	**0.0182**
**3**	226 (52%)	216 (50%)	**0.0460**
**Genetic carrier**	75 (17.2%)	71 (16.3%)	**0.0246**
**Comorbidities**			
**Overweight and obesity**	78 (17.9%)	77 (17.7%)	**0.0060**
**Diabetes mellitus**	33 (7.6%)	32 (7.4%)	**0.0087**
**Cardiovascular disease**	20 (4.6%)	22 (5.1%)	**0.0215**
**Tobacco use**	145 (33.3%)	136 (31.3%)	**0.0443**
**Psychoactive substance use**	21 (4.8%)	20 (4.6%)	**0.0108**
**Receptor status**			
**PR positive**	242 (62.9%)	238 (61.8%)	**0.0214**
**PR negative**	114 (29.6%)	109 (28.3%)	**0.0286**
**HER2 positive**	96 (25%)	86 (22.3%)	**0.0612**
**HER2 negative**	261 (67.8%)	257 (66.8%)	**0.0221**
**Adjuvant therapy**			
**Anastrozole**	33 (7.6%)	33 (7.6%)	**<0.0001**
**Tamoxifen**	14 (3.2%)	14 (3.2%)	**<0.0001**
**Chemotherapy**	336 (77.2%)	330 (75.9%)	**0.0326**
**Radiation**	52 (12%)	52 (12%)	**<0.0001**
**Surgical procedure** (CPT)			
**Partial mastectomy** (19301)	96 (22.1%)	96 (22.1%)	**<0.0001**
**Partial mastectomy with lymphadenectomy** (19302)	0 (0%)	0 (0%)	–
**Simple mastectomy** (19303)	≤10 (2.3%)	≤10 (2.3%)	**<0.0001**
**Mastectomy, subcutaneous** (19304)	0 (0%)	0 (0%)	–
**Radical mastectomy** (19305)	≤10 (2.3%)	≤10 (2.3%)	**<0.0001**
**Radical mastectomy, including internal mammary lymph nodes** (19306)	0 (0%)	0 (0%)	–
**Modified radical mastectomy** (19307)	≤10 (2.3%)	≤10 (2.3%)	**<0.0001**

Bold font represents a standardized difference < 0.1. Abbreviations: Std. diff, standardized difference. *These variables were selected for matching and adjustment and should not be interpreted as a comprehensive description of the overall study population. Due to the longitudinal nature of the TriNetX database, variables are queried independently and are not necessarily mutually exclusive. Percentages reflect the proportion of patients with documented values for each characteristic; therefore, percentages may not sum to 100% due to missing data and incomplete capture within the electronic health record.*

**Table 3:** Comparison of propensity-matched cN1, ER+ breast cancer patients who underwent SLNB vs. ALND.

	SLNB (*n* = 435)	ALND (*n* = 435)	HR (95% CI)
**Mortality, *n* (%)**	68 (15.6%)	76 (17.5%)	0.9 (0.66–1.27)
**Local recurrence of the breast, *n* (%)**	23 (5.3%)	29 (6.7%)	0.8 (0.47–1.42)
**Local recurrence of the axilla, *n* (%)**	93 (21.4%)	105 (24.1%)	0.9 (0.69–1.21)

Bold font indicates a statistically significant value. Abbreviations: 95% CI, 95% confidence interval; HR, hazard ratio.

## Data Availability

The data analyzed in this study were accessed through the TriNetX Network, a federated health research platform that provides de-identified, aggregated patient data from participating healthcare organizations. Access to TriNetX is institution-dependent; in this study, data were accessed through the University of Texas Medical Branch (UTMB). No patient-level data were downloaded, exported, or stored by the authors. Researchers seeking to access similar datasets must request access directly from TriNetX (https://trinetx.com) and comply with both TriNetX data use agreements and their own institutional policies.

## Supplementary Materials

The following supporting information can be downloaded at: https://www.mdpi.com/article/10.3390/cancers18162574/s1, Table S1. Availability and utilization of clinically relevant variables within the TriNetX federated electronic health record network.

## Abbreviations

The following abbreviations are used in this manuscript:

AJCC	American Joint Committee on Cancer
ALND	Axillary lymph node dissection
AMAROS	After Mapping of the Axilla: Radiotherapy Or Surgery? trial
CI	Confidence interval
cN1	Clinically node-positive (axillary lymph node involvement)
CPT	Current Procedural Terminology
ER	Estrogen receptor
ESMO	European Society for Medical Oncology
HER2	Human epidermal growth factor receptor 2
HR	Hazard ratio
ICD-10-CM	International Classification of Diseases, 10th Revision, Clinical Modification
KM	Kaplan–Meier
LR	Local recurrence
NAC	Neoadjuvant chemotherapy/systemic therapy
NCCN	National Comprehensive Cancer Network
NCDB	National Cancer Database
OS	Overall survival
pCR	Pathologic complete response
PR	Progesterone receptor
PSM	Propensity score matching
RNI	Regional nodal irradiation
SLNB	Sentinel lymph node biopsy
SN FNAC	Sentinel Node biopsy Following Neoadjuvant Chemotherapy study
TAXIS	Tailored Axillary Surgery trial
Z1071	ACOSOG Z1071 trial
SENTINA	Sentinel Neoadjuvant trial
