# Supplementary material for: Axillary Management in Clinically Node-Positive (cN1) Estrogen Receptor-Positive Breast Cancer
Source: Cancers (Basel). 2026 Aug 11;18(16):2574. doi: 10.3390/cancers18162574 (PMC13511697; doi:10.3390/cancers18162574)
Supplement: Supplementary file 1 [file cancers-18-02574-s001.zip › cancers-4473779-supplementary.pdf]

Table S1. Availability and utilization of clinically relevant variables within the TriNetX federated electronic health record network.

Variable	Availability in TriNetX	Used in Analysis	Comments
Age	Routinely available	Yes	Core demographic variable available for nearly all patients.
Sex	Routinely available	Yes	Core demographic variable available for nearly all patients.
ER, PR, HER2 status	Routinely available	Yes	Structured biomarker information used for cohort selection and propensity score matching.
Tumor size (T category)	Partially available	Yes	Derived from structured staging documentation when available. Completeness varies across participating healthcare organizations.
Histologic grade	Limited	No	Not consistently available across participating healthcare organizations; therefore, not incorporated into matching.
Chemotherapy	Partially available	Yes	Identified using structured procedure and medication records. Completeness depends on EHR documentation.
Endocrine therapy	Partially available	Yes	Identified using structured medication (RxNorm) records. Absence of documentation should not be interpreted as absence of treatment.
Radiation therapy	Partially available	Yes	Identified using procedure and billing records. Completeness depends on documentation within participating healthcare organizations.
Body mass index (BMI)	Limited	No	BMI values are inconsistently available across healthcare organizations.
Obesity diagnosis	Partially available	Yes	Identified using ICD-10-CM diagnosis codes (e.g., E66); reflects coded diagnoses rather than measured BMI.
Genomic assays (e.g., Oncotype DX recurrence score)	Not reliably available	No	Molecular assay results are not consistently captured across participating healthcare organizations.
Menopausal status	Not reliably available	No	Not reliably captured in structured EHR data; age was used as a surrogate where appropriate.
Sequence of axillary procedures (e.g., completion ALND following SLNB)	Not reliably available	No	The database does not reliably distinguish initial sentinel lymph node biopsy from subsequent completion axillary lymph node dissection. Therefore, the initial documented axillary procedure was used as the index event.
Pattern of recurrence (isolated regional vs. distant metastatic disease)	Not reliably available	No	Administrative diagnosis codes cannot reliably distinguish isolated axillary recurrence from regional or metastatic disease progression.

This table summarizes the availability of clinically relevant variables within the TriNetX federated electronic health record network. Variable availability reflects structured data contributed by participating healthcare organizations and may vary due to differences in documentation and coding practices. Absence of a documented variable should not be interpreted as absence of the corresponding clinical characteristic or treatment.
